# The In Vitro Activity of Fluconazole, Amphotericin B and Echinocandins Against *Cyberlindnera fabianii* Planktonic Cells and Biofilms

**DOI:** 10.1007/s11046-022-00688-9

**Published:** 2022-11-18

**Authors:** Zoltán Tóth, Aliz Bozó, Renátó Kovács, Bettina Balogh, Bence Balázs, Lajos Forgács, Barna Kelentey, László Majoros

**Affiliations:** 1 Department of Medical Microbiology, Faculty of Medicine, University of Debrecen, Nagyerdei Krt. 98., Debrecen, 4032 Hungary; 2 Doctoral School of Pharmaceutical Sciences, University of Debrecen, Debrecen, Hungary; 3 Department of Restorative Dentistry, Faculty of Dentistry, University of Debrecen, Debrecen, Hungary

**Keywords:** Rare fungal pathogen, *Cyberlindnera fabianii*, Susceptibility, Echinocandin, Biofilm, Killing rate

## Abstract

Until recently, little was known about the susceptibility pattern of *Cyberlindnera fabianii* (*Cy. fabianii*) planktonic cells and biofilms regarding the most frequently administered systemic antifungals, despite the high mortality rate and its potential role in catheter-related infections. In the current study, the activity of fluconazole, amphotericin B and echinocandins (anidulafungin, caspofungin and micafungin) was determined against planktonic and sessile cells of *Cy. fabianii* clinical isolates (*n* = 8). Planktonic minimum inhibitory concentrations (MICs) ranged from 1 to 2, from 0.25 to 1, from 0.015 to 0.06, from 0.03 to 0.12 and from 0.25 to 0.5 mg/l for fluconazole, amphotericin B, anidulafungin, caspofungin and micafungin, respectively. One-day-old biofilms were highly resistant to fluconazole (MIC ranged from 512 to > 512) compared to planktonic counterparts, but not to amphotericin B (MIC ranged from 0.25 to 2 mg/l) and echinocandins (MIC ranged from 0.06 to 2 mg/l). Based on the calculated planktonic killing rates, the highest activity was observed in the case of anidulafungin (*k* values ranged from 0.37 to 2.09), while micafungin, caspofungin, amphotericin B and fluconazole exerted 0.46–1.47, 0.14–0.86, −0.03 to 2.08 and −0.15 to 0.09 killing rate value ranges, respectively. The obtained in vitro planktonic and sessile susceptibility patterns suggest that echinocandins and amphotericin B may be the most reliable treatment option for the treatment of *Cy. fabianii* infections.

## Introduction

*Cy. fabianii* is a rarely isolated pathogenic yeast, which is associated with endocarditis, prostatitis and fungaemia as well as catheter-related infections. In the past decade, the emergence of *Cy. fabianii* is steadily growing, presumably due to the increasingly usage of indwelling devices [1, 2]. The virulence attributes of *Cy. fabianii* are obscure similarly to its prevalence; however, biofilm formation is implicated as an important factor in the pathogenesis of this species [3, 4]. Nevertheless, this fungal species is considered to have low virulence [5]. In spite of this relatively low virulence, *Cy. fabianii* may cause severe life-threatening infections, which are associated with an almost 25% mortality rate [3]. In addition, hospital outbreaks have been also reported in pre-term newborns, suggesting the emergence of nosocomial transmission [6]. The number of described predisposing factors is scarce, but severe immunosuppression, low birth weight and surgical intervention may contribute to the infection [7]. It is noteworthy that the usage of indwelling catheters is one of the most important sources of infections, further emphasising the role of biofilm development in *Cy. fabianii-*related cases [1, 7]. The optimal therapeutic strategy against *Cy. fabianii* is not yet well defined because acquired resistance can be observed against frequently administered antifungals [4, 8, 9]. However, pharmacodynamic analysis has a pivotal role in therapy optimisation; until recently, little was known about the susceptibility to and killing rate of the most frequently used antifungal drugs against *Cy. fabianii*. Park et al. [1] reported low minimum inhibitory concentration (MIC) values for echinocandins and amphotericin B similar to *Candida albicans*, while MICs to fluconazole values were elevated (1–4 mg/l). To expand the therapeutic options in clinical practice against *Cy. fabianii*, the in vitro pharmacodynamic properties of fluconazole, amphotericin B, anidulafungin, caspofungin and micafungin against this rare fungal species were determined.

## Materials and Methods

### Isolates

All isolates (*n* = 8) except 21,605 were identified and collected at the University of Debrecen, Department of Medical Microbiology between 2019 and 2021 from various anatomical sites (blood, urine, pharynx, nephrostomy catheter) (Table 1). Identification was performed with Matrix Assisted Laser Desorption/Ionisation Time of Flight mass spectrometry using a Bruker Biotyper instrument (Bruker, Bremen, Germany) according to the manufacturer’s instructions. Species level identification was accepted if the score was ≥ 2. Isolate 21,605 was derived from a previous study [10]. The isolates were stored at −70 °C until the experiments.

**Table 1:** MIC values of fluconazole (FLU), amphotericin B (AMB) anidulafungin (ANF), micafungin (MCF) and caspofungin (CSF) in RPMI-1640 against *Cy. fabianii* clinical isolates. MIC determination was performed according to CLSI M27 ed4. in duplicates

Isolate	Anatomical site	MIC (mg/l)				
		FLU	AMB	ANF	MCF	CSF
45,565	Blood	1/2	0.5	0.03	0.06	0.5
41,785	Pharynx	1	0.5	0.015	0.03	0.25
34,271	Urine	1	0.5	0.03	0.03	0.25
38,360	Bronchus	1	0.5–1	0.03/0.06	0.03	0.5
21,605	Nephrostomy catheter	1	0.5	0.06	0.12	0.5
41,852	Urine	1	0.25	0.03	0.06	0.25
48,766	Blood	2	0.25	0.03	0.03	0.25/0.5
42,268	Bronchus	1/2	0.25–0.5	0.03	0.03	0.25

### Antifungal Susceptibility Testing

Antifungal susceptibility testing was performed according to the CLSI M27 ed.4 guideline in RPMI-1640 (with L-glutamine and without bicarbonate, pH 7.0 and with MOPS; Merck, Budapest, Hungary) supplemented with 2% glucose (Sigma-Aldrich) [11]. Anidulafungin, caspofungin and micafungin pure powders were obtained from MolCan (Toronto, Canada), while fluconazole and amphotericin B pure powders were purchased from Sigma-Aldrich (Budapest, Hungary). The MICs were determined as the lowest antifungal concentration that exerts at least 50% growth inhibition compared to the untreated growth control for echinocandins and fluconazole. In the case of amphotericin B, 100% growth inhibition was considered the MIC value. *C. parapsilosis* ATCC 22,019 and *C. krusei* ATCC 6258 were used as quality control strains. The experiments were performed in triplicate. As clinical breakpoints for *Cy. fabianii* are not yet defined, only the MIC values are reported.

### Time-Kill Studies

Determination of the killing activity of each agent tested was performed in RPMI-1640 medium in a total volume of 10 ml using time-kill methodology, as described previously [12]. The tested concentrations ranged from 0.25 to 8 mg/l for fluconazole and echinocandins and from 0.25 to 2 mg/l in the case of amphotericin B. Concentrations were defined according to the published pharmacokinetic parameters of each antifungal agent during therapy with standard dosages [13, 14]. The starting inoculum was 1–1.2 × 10^5^ CFU/ml (Colony Forming Unit per millilitre). At 0, 4, 8, 12 and 24 h of incubation, 100 µl aliquots were serially diluted and then 4 × 30 µl of each dilution were plated onto Sabouraud Dextrose Agar supplemented with chloramphenicol (SDA). The SDA plates were incubated for 48 h at 37 °C before colony counting.

### Analysis of Time-Kill Results

In vitro killing activity was expressed by calculating the killing rate values for each isolate and antifungal concentration combinations by fitting the following exponential equation on data points: N_t_ = N_0_*e^−kt^, where N*t* is the number of viable cells at a given timepoint, *N*_*0*_ is the starting inoculum, e is the Euler’s number, *t* is the elapsed time in hours and *k* is the killing rate. Positive *k* values indicate a decrease in the living cell numbers while negative *k* values indicate growth. The goodness of fit was tested using the r^2^ test (r^2^ ≥  ± 0.8). The killing activity of the tested antifungal agents for each isolate was compared using one-way ANOVA (with Tukey’s post-hoc test). The time needed to achieve a fungicidal (99.9% CFU decrease) endpoint was calculated as follows: T_99.9_ = 3/*k *[12].

### In Vitro* Biofilm Formation*

The biofilm forming ability of the *Cy. fabianii* isolates was assessed using a crystal-violet assay (CV assay) as described earlier [15]. Briefly, two-day-old fungal cells were harvested from solid SDA plates by centrifugation (3000 g for 5 min), washed three times in sterile physiological saline and then the final density of the inocula was adjusted in RPMI-1640 broth to 1 × 10^6^ cells/ml. Afterwards, 100 µl aliquots were inoculated onto flat-bottom 96-well sterile microtitre plates (TPP, Trasadingen, Switzerland) and incubated at 37 °C for 24 h in the dark. As there are no data available to categorise *Cy. fabianii* isolates based on their biofilm forming ability, the *C. albicans* SC5314 reference strain was included in the experiments to compare the obtained CV results. Absorbance was measured at 540 nm using a Thermo-Fisher MultiSKAN Sky plate reader. The experiments were performed in triplicate and the median values are shown. Statistical comparison was performed using one-way ANOVA with Dunnett’s post-hoc test.

### Sessile Population Susceptibility Testing

The susceptibility of one-day-old biofilms was tested after a further 24-h incubation in RPMI-1640 containing various concentrations of antifungals. The tested concentrations ranged from 0.008 to 2 mg/l, from 0.06 to 16 mg/l and from 1 to 512 mg/l for echinocandins, amphotericin B and fluconazole, respectively. The biofilm MICs were defined as the lowest drug concentration resulting in at least a 50% reduction in metabolic activity compared with unexposed controls and are presented as the median value of three independent experiments per isolate. Metabolic activity was quantified by the XTT [2,3-bis (2-methoxy- 4-nitro-5-sulphophenyl)-2H-tetrazolium-5-carboxanilide] assay, as described previously [16]. Antifungal agents were removed prior assaying metabolic activity by washing with physiological saline. Afterwards, a 100 µl aliquot of XTT solution was added to each well containing the pre-washed sessile cells. Then, plates were covered with aluminium foil and incubated in the dark for 2 h at 37 °C. Following incubation, 80 µl of supernatant from each well was measured photometrically at 492 nm (Thermo-Fisher MultiSKAN Sky plate reader). Experiments were performed in triplicate and the median sessile MICs are shown.

## Results

### MIC Values of the Cy. fabianii Isolates

The MIC values for *C. krusei* ATCC 6258 and *C. parapsilosis* ATCC 22,019 were within the accepted quality control (QC) ranges for all tested antifungals [17]. There are no clinical breakpoints available for *Cy. fabianii*; nevertheless, according to the epidemiological cut-off values (ECVs) published by Lee et al. [18], all tested isolates had an MIC above the ECV for fluconazole (ECV 0.5 mg/l), 3 out of 8 (21,605, 45,565, 34,271) for micafungin (ECV: 0.03 mg/l) and none for amphotericin B (ECV: 2 mg/l) (Table 1). The lowest MIC values were observed in the case of anidulafungin (ranged from 0.015 to 0.06 mg/l) and micafungin (ranged from 0.03 to 0.12 mg/l), while the MIC values to caspofungin ranged from 0.25 to 0.5 mg/l, similar to amphotericin B. Fluconazole MICs ranged from 1 to 2 mg/l (Table 1).

**Fig. 1:**
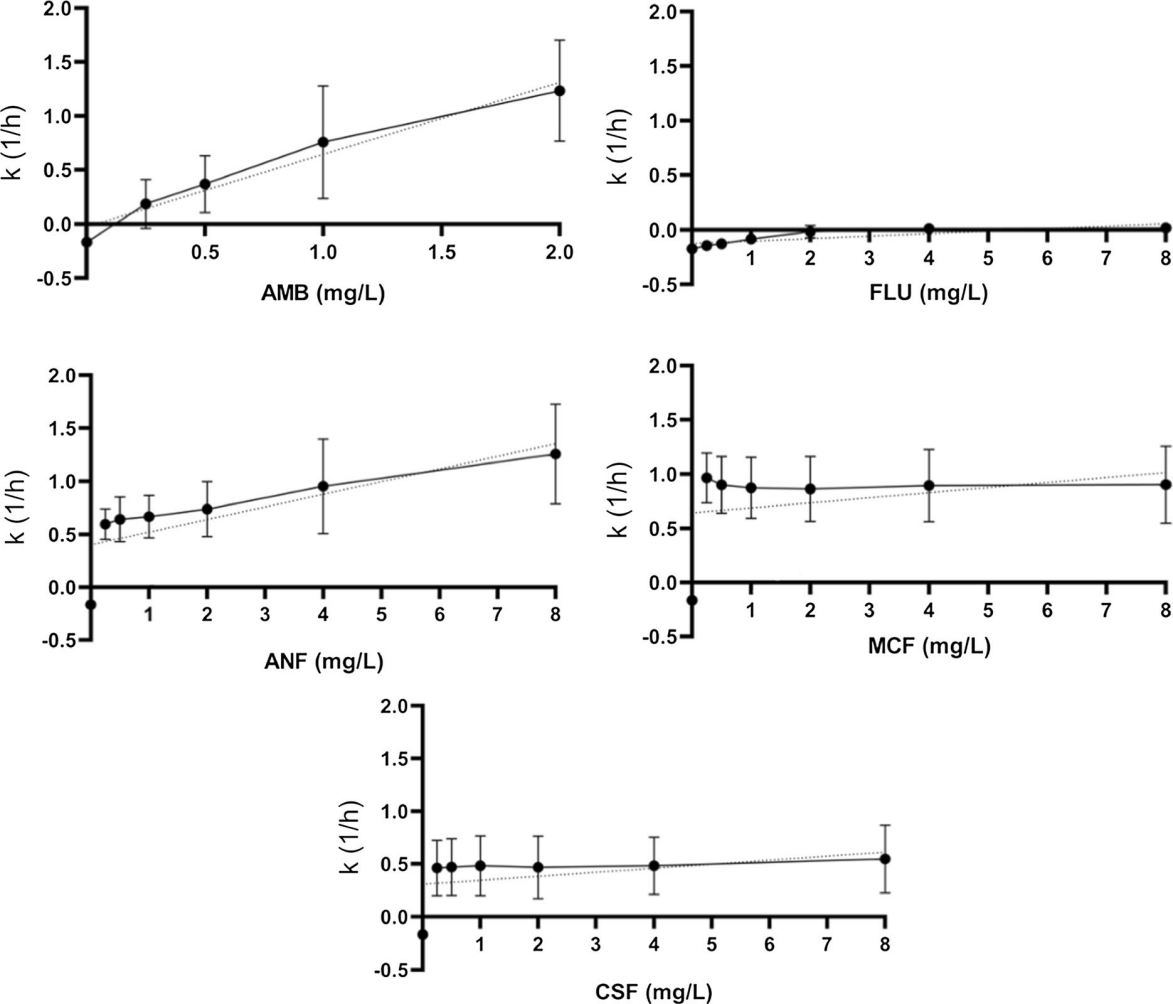
Mean killing rate values of amphotericin B (AMB), fluconazole (FLU), anidulafungin (ANF), micafungin (MCF) and caspofungin (CSF) in RPMI-1640 against *Cy. fabianii* isolates. Positive and negative *k* values indicate the decrease and increase, respectively, in viable cell numbers. Error bars represent standard deviation (SD). The dotted line is a linear regression fitted on the observed *k* values, apart from drug-free control

### Results of the Time-Kill Experiments

The in vitro activity of echinocandins was comparable against tested isolates. However, micafungin and caspofungin showed a concentration-independent killing effect, while anidulafungin exerted significantly higher activity at 8 mg/l (average 1/*k *was 1.25) compared to the effect observed at concentrations ranging from 0.25–1 mg/l (average 1/*k *values were 0.58, 0.64 and 0.66 respectively) (Fig. 1). Eight mg/l of anidulafungin exposure resulted in a notably shorter time required to achieve fungicidal effects compared to the lower concentrations (0.25–1 mg/l) (Table 2). T99.9 values were not significantly different in the case of caspofungin and micafungin at the examined concentrations (Table 2).

**Fig. 2:**
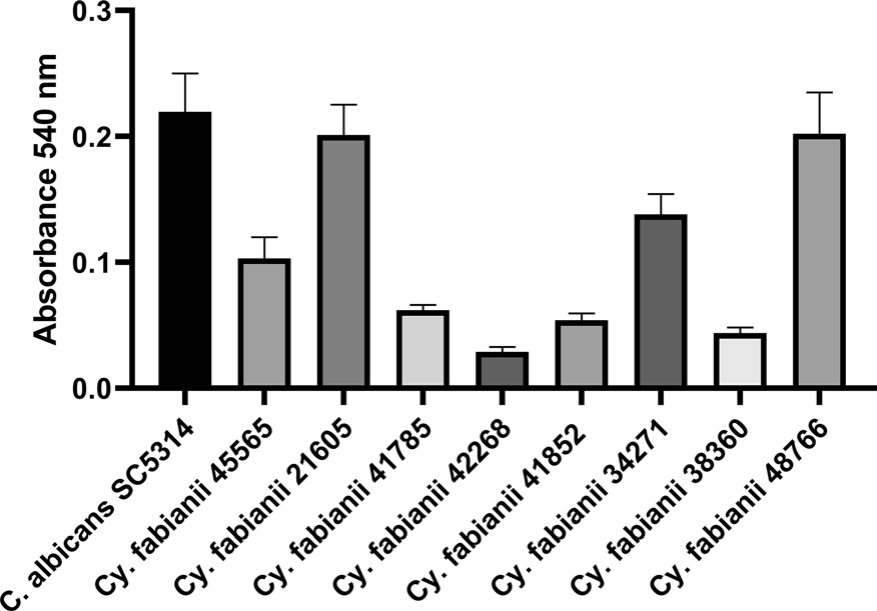
Average absorbance values at 540 nm for the *Cy. fabianii* clinical isolates and the SC5314 *C. albicans* strain using crystal-violet assay. The experiments were performed in triplicates, the error bars show the standard deviation

**Table 2:** Mean time (hours) ± SD to reach 99.9% (T_99.9_) growth reduction (T_99.9_ = 3/*k*) from the starting cell numbers at different, anidulafungin (ANF), micafungin (MCF), caspofungin (CSF) and amphotericin B (AMB) concentrations in RPMI-1640. NA: 99.9% growth inhibition was not achieved for any of the isolates; *ND* not determined

Drugs	T_99.9_					
	8 mg/l	4 mg/l	2 mg/l	1 mg/l	0.5 mg/l	0.25 mg/l
ANF	2.69 ± 0.98	3.69 ± 1.44	4.42 ± 1.26	4.91 ± 1.7	5.05 ± 1.39	5.28 ± 1.31
MCF	3.82 ± 1.54	3.79 ± 1.42	3.88 ± 1.43	3.76 ± 1.24	3.63 ± 1.25	3.29 ± 0.92
CSF	7.22 ± 3.89	8.13 ± 4.41	8.58 ± 4.7	8.79 ± 5.99	9.65 ± 7.36	NA
AMB	ND	ND	2.77 ± 1.11	5.72 ± 3.72	13.05 ± 10.27	NA
FLU	NA	NA	NA	NA	NA	NA

Micafungin and anidulafungin produced fungicidal effects after 24 h at all of the tested concentrations against seven isolates, except for 41,852 and 21,605 for micafungin and anidulafungin, respectively. In these two cases, only a 99% reduction in CFU number was observed (fungistatic effect). Fungicidal effects were observed with caspofungin above 0.25 mg/l in the case of six isolates, except for isolates 21,605 and 41,785. It is noteworthy that a pronounced “mini-paradoxical” effect was observed for these two isolates in response to caspofungin exposure (*k* values gradually decreased from 0.45 to 0.8 1/*k* at 0.25 mg/l to 0.29 and 0.46 1/*k* at 8 mg/l respectively), while the average *k* value was slightly higher in the case of micafungin at 0.25 mg/l (0.96 average 1/*k*) compared to the *k* values obtained at the higher concentrations tested (0.89 and 0.9 average 1/*k* at 4 and mg/l respectively) (*p* > 0.05) (Fig. 1, Table 2).

**Table 3:** The median sessile MIC values observed in case of fluconazole (FLU), amphotericin B (AMB), anidulafungin (ANF), micafungin (MCF) and caspofungin (CSF) against one day old mature biofilms of *Cy. fabianii* clinical isolates

Isolates	Median sessile MIC values (mg/l)				
	FLU	AMB	ANF	MCF	CSF
45,565	> 512	1	2	0.125	0.5
21,605	> 512	2	0.25	0.5	0.25
41,785	> 512	1	2	0.5	1
41,852	> 512	0.5	2	0.125	2
34,271	> 512	0.25	0.125	0.25	0.5
38,360	> 512	0.5	0.06	0.125	0.25
48,766	> 512	0.5	0.06	0.25	0.5
42,268	512	2	0.125	0.125	2

Two mg/l of amphotericin B exerted a higher killing rate compared to 0.25 mg/l (avg. 1/*k* was 1.23 vs. 0.18 respectively), showing concentration-dependent activity; however, this effect was strongly isolate-dependent. Growth was observed at 0.25 mg/l in the cases of isolates 41,852 and 21,605, while a weak fungistatic effect was detected against six of the eight strains. Surprisingly, 2 mg/l was not fungicidal against isolates 41,852 and 42,268 after 24 h of exposure, while 1 mg/l exerted the fungicidal endpoint in the case of the other six isolates. The average time necessary to achieve the fungicidal endpoint for amphotericin B at 2 mg/l was similar to anidulafungin at 8 mg/l. As expected, fluconazole had a fungistatic activity between 2 mg/l and 8 mg/l against all isolates and achieved a 50% CFU decrease against only two of the eight isolates (21,605 and 45,565) at the highest tested concentration (8 mg/l).

### Assessment of Biofilm Forming Ability and Sessile Susceptibility to Antifungals

Interestingly two *Cy. fabianii* isolates (21,605 and 48,766) proved to be as good biofilm formers compared to the SC5314 *C. albicans* reference strain in our experimental setting (Fig. 2). In contrast, the biofilm mass of the other six isolates was significantly lower compared to *C. albicans* biofilm (*p* < 0.005). Overall, micafungin showed the highest anti-biofilm effect with a median biofilm MIC ranging from 0.125 to 0.5 mg/l. In contrast, the two other echinocandins produced a weaker efficacy, where median sessile MICs ranged from 0.25 to 2 mg/l and from 0.06 to 2 mg/l for caspofungin and anidulafungin, respectively. Fluconazole had a negligible effect on sessile populations with an average median sessile MIC of > 512 mg/l. In the case of fluconazole, a 90% decrease in metabolic activity was not achieved after 24 h for any of the isolates at the tested concentration ranges (Table 3).

## Discussion

Over the past few decades, the prevalence of infections caused by rare *Candida* species has shown a significant increase [19, 20]. Although the true frequency of its prevalence is unknown, *Cy. fabianii* may be more prevalent than was previously reported [1, 2]. Based on the limited clinical data available, *Cy. fabianii* does not show intrinsic resistance to any antifungal agents; however, the most appropriate therapeutic approach is still unclear and, despite its low virulence, therapeutic failures have been noticed with various antifungal regimens [3, 5]. This worrisome trend highlights the need for a better understanding of the killing pattern of the currently approved antifungal drugs against this emerging opportunistic pathogen. To expand our knowledge, we have assessed the activity of fluconazole, amphotericin B and three licensed echinocandins against *Cy. fabianii* clinical isolates. The susceptibility results were concordant with previous reports published by Park et al. [1] Time-kill studies revealed a concentration-independent fungicidal effect for caspofungin and micafungin at concentrations from 0.25–8 mg/l; however, the activity of anidulafungin was concentration-dependent. The shortest time needed to achieve fungicidal activity was also observed for the latter (Table 2). Of the currently approved echinocandins, anidulafungin is reported to have the highest in vitro activity against various *Candida* species, but this difference is often diminished compared to micafungin and caspofungin in the presence of serum due to their different protein binding properties [21]. Amphotericin B also showed a concentration-dependent fungicidal activity; however, this effect was strain-dependent and had no fungicidal effect against two isolates, even at a concentration of 2 mg/l. Contrastingly, fluconazole had a fungistatic activity above the respective MICs of the isolates, which is also similar to the observed activity against commonly isolated *Candida* spp. [22]. The lack of data makes the comparison of time-kill results impossible; however, all antifungals tested produced good activity against planktonic cells at clinically attainable peak concentrations in the serum (C_max_ is 9 mg/l, 2 mg/l, 10 mg/l, 7 mg/l and 9 mg/l with standard dosages of fluconazole, amphotericin B, caspofungin, micafungin and anidulafungin, respectively) [13, 14]. The lack of a fungicidal effect of amphotericin B and echinocandins against certain isolates indicates that their activity may vary against *Cy. fabianii,* even though they had susceptible phenotype by standard susceptibility testing. While the biofilm-forming ability of *Cy. fabianii* isolates presumably played a pivotal role in the reported clinical cases, there are no data on the activity of antifungals against biofilms formed by this species to date. Based on several epidemiological studies, the biofilm formation is associated with higher mortality rates in cases of other rare *Candida* species [23, 24]. Of the isolates tested in our experiments, two proved to be excellent biofilm formers compared to the SC5314 *C. albicans* reference strain. The activity of the tested antifungals against sessile populations was more diverse compared to the planktonic populations. Based on the obtained MIC values, echinocandins and amphotericin B had comparable activity and that similar to the sessile susceptibility profile of *C. kefyr* [16]. In contrast, a marked difference was observed in the case of fluconazole between the planktonic and sessile populations. While 2 mg/l of fluconazole had an inhibitory effect against all of the tested isolates in the experiments involving planktonic cells, the median MIC value was > 512 mg/l. The weak efficacy of fluconazole against sessile *Cy. fabianii* cells is in line with previous studies suggesting that fluconazole is not a biofilm active agent against other *Candida* spp. [25]. Whether that poor anti-biofilm activity may have played a role in the therapeutic failures during fluconazole treatment has not yet been proven for *Cy. fabianii*; nonetheless, a similar mechanism is implicated in the case of more common *Candida* isolates [26].

To the best of our knowledge, this is the first comprehensive study on the in vitro pharmacodynamic properties of the most frequently used antifungal agents against *Cy. fabianii*. Our results suggest that echinocandins are a reasonable first-line agents against this species. The activity of amphotericin B against planktonic and sessile populations was also high, but the associated toxicity with the conventional amphotericin B-deoxycholate and the high cost of newer formulations may limit its therapeutic usage for the adult population. In the future, further comparative in vivo experiments are needed to confirm whether these in vitro results have clinical relevance in the treatment of *Cy. fabianii* infections.
